# Sub-annular composite graft implantation for infective endocarditis

**DOI:** 10.1007/s12055-023-01544-1

**Published:** 2023-06-12

**Authors:** Pietro Giorgio Malvindi, Michele Galeazzi, Paolo Berretta, Olimpia Bifulco, Beatrice Buratto, Mariano Cefarelli, Jacopo Alfonsi, Alessandro D’Alfonso, Marco Di Eusanio

**Affiliations:** 1 Ospedali Riuniti, Polytechnic University of Marche, Ancona, Italy; 2 Cardiac Surgery Unit, Lancisi Cardiovascular Center, Ancona, Italy; 3 Cardiac Surgery Unit, Department of Cardiac, Thoracic, Vascular Sciences and Public Health, University of Padua, Padua, Italy

**Keywords:** Subannular Bentall, Endocarditis, Pseudoaneurysm

Surgical treatment of infective endocarditis with major disruption of the aortic valve annulus and involvement of the mitral-aortic curtain and the aortic root is a great challenge [1, 2]. Here we report our technique for root replacement with sub-annular implantation of a composite graft. It involves [1] aggressive debridement of the infective tissue with extensive isolation and mobilization of the aortic root and coronary ostia, and [2] proximal suture line for composite graft implantation running below the abscess on the interventricular septum. This allows the abscess to drain freely into the pericardium.

The patient was a 66-year-old male with prosthetic aortic valve endocarditis. Preoperative transesophageal echocardiography showed a large pseudoaneurysm of the mitral-aortic intervalvular fibrosa.

After median re-sternotomy, cardiopulmonary bypass (CPB) institution was obtained by central cannulation (aortic arch and venous bicaval drainage). Cold crystalloid cardioplegia was administered, the aortotomy was performed and the aortic root isolated. The bioprosthesis was removed and the coronary buttons were prepared. The aortic root was resected and a composite graft (23-mm Edwards Magna aortic valve and 28-mm Terumo Valsalva graft) was chosen. The proximal suture line was identified at the lower edge of the abscess, on the left ventricular outflow tract. A running suture with bites including the muscular septum and a small portion of the anterior mitral leaflet was performed. The coronary ostia were reimplanted and the distal anastomosis between the composite graft and the aorta was completed. The operation was concluded in the usual fashion.

Main advantages of the presented technique consist of excluding the abscess completely and avoiding tension on surrounding tissues (septum, anterior mitral leaflet), thus reducing the risk of suture dehiscence.

In our institution, 25 high-risk patients have undergone this kind of intervention since late 2016, all of them being reoperations. Two patients died, one due to multi-organ failure and one another due to cerebral bleeding, with an overall 30-day mortality of 9%. Considering this high-risk profile cohort, the preliminary results are encouraging and suggest safety and efficacy of this approach. Nonetheless, we are aware more robust data are still needed.

### Supplementary Information

ESM 1 (MP4 196546 kb)

## Data Availability

The data that support the findings of this study are available on request from the corresponding author. The data are not publicly available due to privacy or ethical restrictions.
